# Expression of kiwifruit-derived actinidin in *Nicotiana benthamiana* leaves

**DOI:** 10.3389/fpls.2024.1532170

**Published:** 2025-01-10

**Authors:** Ji Hyun Kang, Jae-Ho Lee, Dong Wook Lee

**Affiliations:** ^1^ Department of Integrative Food, Bioscience and Biotechnology, Chonnam National University, Gwangju, Republic of Korea; ^2^ Department of Bioenergy Science and Technology, Chonnam National University, Gwangju, Republic of Korea; ^3^ Bio-Energy Research Center, Chonnam National University, Gwangju, Republic of Korea

**Keywords:** actinidin, molecular farming, protein expression, protein purification, β-casein degradation

## Abstract

Kiwifruit (*Actinidia deliciosa*)-derived actinidin, a cysteine protease, is renowned for its meat-tenderizing and milk-clotting activities. Despite its potential in various biotechnological applications, an efficient expression platform for actinidin production has not yet been developed. Instead, actinidin has traditionally been purified directly from the fruits of various plants. This study aimed to produce kiwifruit-derived actinidin in the leaves of *Nicotiana benthamiana*. The expressed actinidin was directed to the lumen of the endoplasmic reticulum (ER) using the binding immunoglobulin protein (BiP) signal sequence and an ER retention signal. To facilitate cost-effective purification, the family 3 cellulose-binding module (CBM3) was employed as an affinity tag, along with microcrystalline cellulose beads that bind efficiently to CBM3. A significant portion of the expressed actinidin was recovered in the soluble fraction without proteolytic degradation. The purified actinidin exhibited β-casein-degrading activity, with optimal efficiency observed at 55°C and pH 7.0. These results establish a promising plant-based platform for the efficient production and functional application of kiwifruit-derived actinidin in diverse biotechnological processes.

## Introduction

Actinidin (EC 3.4.22.14) is a cysteine protease found in various fruits, including kiwifruit (*Actinidia deliciosa*), mango, pineapple, and papaya. Kiwifruit-derived actinidin, in particular, is widely recognized for its applications in meat tenderization and milk clotting, making it a valuable tool in the food industry (Aminlari et al., 2009; Katsaros et al., 2010; Nicosia et al., 2022; Abril et al., 2023; Mohd Azmi et al., 2023). Despite its potential, active actinidin is still primarily extracted and purified directly from kiwifruit (Nam et al., 2006; Wang et al., 2016; Maskey and Karki, 2024). Previous attempts to express actinidin derived from Chinese wild kiwifruit in *Escherichia coli* resulted in the formation of inclusion bodies, necessitating additional refolding steps to obtain active protein (Lee and Hahm, 2007). Consequently, despite its numerous applications, the commercial utilization of actinidin remains limited due to the lack of an efficient and scalable production platform.

Plant molecular farming, the production of valuable proteins in plants, has emerged as a promising strategy to overcome the limitations of traditional protein production systems (Buyel, 2019; Schillberg and Finnern, 2021; Jadhav and Khare, 2024). This approach offers several advantages, including cost-effectiveness, scalability, and safety, as plants are free from animal pathogens or endotoxins from *E. coli*. Among various plant species, *Nicotiana benthamiana*, a close relative of tobacco, has become a popular host for recombinant protein expression due to its rapid growth, ease of genetic manipulation, and high biomass yield (Bally et al., 2018; Ranawaka et al., 2023; Vollheyde et al., 2023). Moreover, using *N. benthamiana* as biofactories not only reduces production costs but also provides a sustainable and environmentally friendly alternative because as a plant-based system, *N. benthamiana* does not involve the use of harmful chemicals, such as the ones required for mammalian or insect cell cultures, and produces fewer waste products. Notably, transient expression systems have emerged as a highly efficient and versatile tool in plant molecular farming. One of the primary benefits is the speed of protein production. Unlike stable expression systems, which can take months to generate transgenic plants and produce the target protein, transient expression systems allow for rapid production within days or weeks. In addition, transient expression systems are less susceptible to the problem of gene silencing, which often occurs in stable transgenic plants (Vaucheret et al., 1998; Rajeevkumar et al., 2015).

In this study, we explored the potential of *N. benthamiana* as a plant-based expression system for producing kiwifruit-derived actinidin. The expressed actinidin was directed to the endoplasmic reticulum (ER) lumen using a BiP signal sequence and an ER retention signal to enhance its stability and accumulation. To facilitate purification, we utilized cellulose-binding module 3 (CBM3), achieving a high yield of active enzyme. The functionality of the plant-produced actinidin was validated through its ability to degrade β-casein as a substrate. These findings underscore the feasibility of using *N. benthamiana* as a sustainable platform for producing functional actinidin, paving the way for its industrial applications.

## Materials and methods

### Plant materials and growth conditions

*N. benthamiana* plants were cultivated in soil under greenhouse conditions maintained at 23–24°C with 40–65% relative humidity and a 16-hour light/8-hour dark photoperiod. Leaves from 6- to 7-week-old plants were harvested for agro-infiltration experiments.

### Plasmid DNA construction

Actinidin is a vacuolar protein containing an N-terminal hydrophobic signal peptide required for cotranslational translocation into the ER lumen (Paul et al., 1995). In this study, we expressed the actinidin sequence (amino acids 25–381) without the N-terminal signal peptide, as this hydrophobic region could affect the solubility of the expressed protein. The GenBank accession number for the actinidin used in this study is MT463287. To generate the construct *BiP-M-CBM3-bdSUMO-Actinidin-HA-HDEL* (*MCS-Actinidin*), the sequence for *Actinidin-HA-HDEL* was synthesized (BIONICS Co., Ltd. Korea). A NaeI restriction site and a GGA codon for Gly were added at the 5′ end, while sequences coding for the HA tag, HDEL ER retention signal, TAA stop codon, and XhoI restriction site were added at the 3′ end. The synthesized *actinidin-HA-HDEL* was digested with NaeI and XhoI restriction endonucleases and ligated into a pUC-based vector pre-digested with the same enzymes. The pUC-based vector used in this study contained sequences coding for the BiP signal sequence, the M-domain of the human receptor-type tyrosine-protein phosphatase C, family 3 cellulose-binding module (CBM3), the small ubiquitin-like modifier (bdSUMO) cleavage site of *Brachypodium distachyo*n, and the HSP transcriptional terminator (Nagaya et al., 2010; Islam et al., 2019). The resultant plasmid was further digested with XbaI and EcoRI and ligated into the pCambia 1300 binary vector, which had been digested with the same restriction enzymes. All restriction endonucleases were purchased from New England Biolabs (Ipswich, MA, USA).

### Agro-infiltration of *MCS-Actinidin* into the leaves of *N. benthamiana*

The *MCS-Actinidin* construct was introduced into *Agrobacterium tumefaciens* (EHA105) via electroporation. The transformed *A. tumefaciens* cells were subsequently infiltrated into *N. benthamiana* leaves using syringe infiltration (Islam et al., 2019). During each agro-infiltration procedure, *A. tumefaciens* carrying the *p38* gene, derived from the Turnip crinkle virus and encoding a suppressor of host gene silencing, was co-transformed to enhance expression.

### Purification of actinidin from *N. benthamiana* leaves

The purification of the expressed actinidin was performed as previously described (Islam et al., 2019; Zaikova et al., 2022; Lee et al., 2024). Frozen leaf tissues (10 g fresh weight), harvested on days 3, 5 and 7 after agro-infiltration, were ground in liquid nitrogen. Total protein extracts were prepared by homogenizing the ground leaves in 30 mL of protein extraction buffer (50 mM Tris–HCl, pH 7.5, 150 mM NaCl, 1 mM DTT, 1% (v/v) Triton X-100, and 1× EDTA-free protease inhibitor cocktail (Roche, Switzerland)). After incubation at 4°C for 15 min, the extracts were filtered through Miracloth (Merck Millipore, USA). A 100 μL aliquot of the total extract was collected as the total (T) fraction. The remaining extracts were centrifuged at 19,400 ×g for 15 min at 4°C, and a 100 μL aliquot of the supernatant was collected as the soluble (S) fraction. The pellets were resuspended in 30 mL of protein extraction buffer, and a 100 μL aliquot was collected as the pellet (P) fraction. The soluble (S) fraction was used for purifying MCS-Actinidin with microcrystalline cellulose (MCC) beads (Sigma-Aldrich, St. Louis, MO, USA; CAS Number 9004-34-6) as described previously (Islam et al., 2019). Following MCC bead-based purification, the N-terminal domains containing CBM3 and bdSUMO were removed using His-bdSENP1 protease, which had been separately purified from *E. coli* BL21 (DE3) (Islam et al., 2019). All protein samples were analyzed by SDS-PAGE, followed by Coomassie Brilliant Blue (CBB) (BioShop, Canada; CAS Number 6104-59-2) staining or Western blotting using an anti-HA antibody (Roche, Basel, Switzerland; CAS Number 11867423001).

### Western blot analysis

Western blotting was performed as described previously (Geem et al., 2021).

### Degradation of β-casein by actinidin expressed in *N. benthamiana*

The purified actinidin was dialyzed against 0.1 M Tris-HCl, pH 7.0. Separately, 0.1 mg of β-casein (Merck & Co., Inc., USA) was dissolved in 0.1 M Tris-HCl, pH 7.0. Dialysis was carried out using Slide-A-Lyzer Dialysis Cassettes with a molecular weight cutoff of 10 kilodaltons (Thermo Scientific, catalog #66380). After dialysis, the concentration of actinidin was determined using a bicinchoninic acid assay kit (Abbkine, catalog no. KTD 3001, Wuhan, China). Subsequently, the yield of produced actinidin per biomass was calculated based on the initial mass of transformed leaves and the concentration of purified actinidin. Then, 7 μg of β-casein was incubated with 70 ng of the purified actinidin at the indicated temperatures and durations. The degradation of β-casein was analyzed by SDS-PAGE, followed by CBB staining. The band intensity of β-casein was quantified using ImageJ software (National Institutes of Health, USA).

## Results

### Construct design for the expression of MCS-Actinidin in *N. benthamiana* leaves

**Figure 1:**
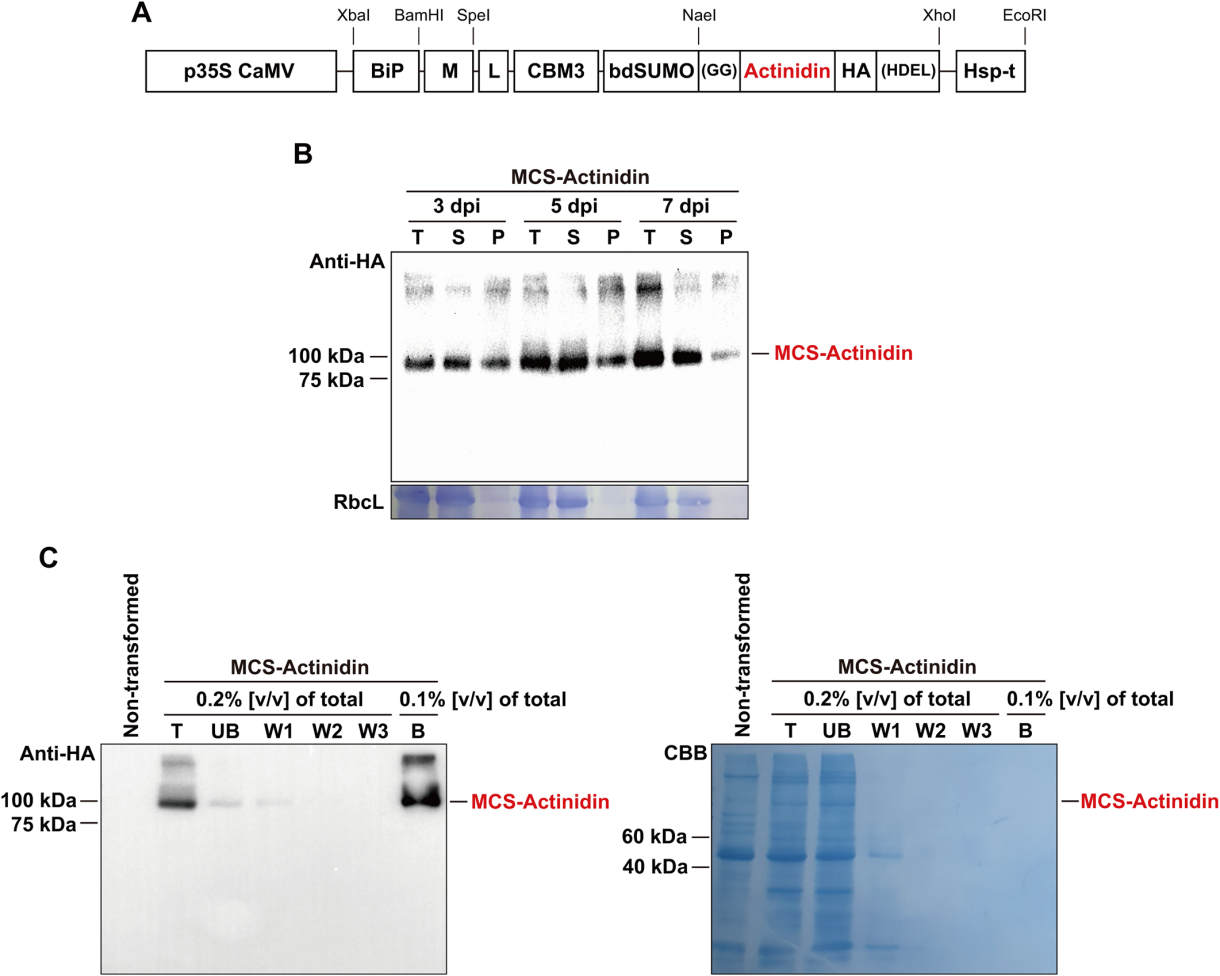
Purification of MCS-Actinidin expressed in *N. benthamiana* leaves. **(A)** Schematic representation of *MCS-Actinidin* construct. BiP, the signal sequence of BiP; M, extracellular domain (amino acid residues 231–290) of human protein tyrosine phosphatase receptor type C; CBM3, cellulose-binding module 3 of *Clostridium thermocellum*; bdSUMO, SUMO domain of *Brachypodium distachyon*; HDEL, ER retention signal; L, flexible linker. **(B)** Expression of *MCS-Actinidin* in the leaves of *N. benthamiana*. Leaves infiltrated with *MCS-Actinidin* were harvested at 3, 5, and 7 days post-infiltration (dpi). Total protein extracts were subjected to centrifugation at 19,400 ×g for 10 minutes. The total (T), soluble (S), and pellet (P) fractions were analyzed by Western blotting using an anti-HA antibody. Proteins were resolved on a 10% SDS-PAGE gel. RbcL: large subunit of the Rubisco complex stained with Coomassie Brilliant Blue (CBB). **(C)** Affinity purification of MCS-Actinidin from *N. benthamiana* leaves. The soluble fraction from **(B)** was incubated with MCC beads. After centrifugation, the unbound fraction (UB) was collected. The MCC beads containing MCS-Actinidin were washed three times (W1–W3). MCS-Actinidin bound to MCC beads **(B)** was eluted by boiling in protein sample buffer. Each fraction was analyzed by Western blotting using an anti-HA antibody. Proteins were resolved on a 10% SDS-PAGE gel. CBB, Coomassie Brilliant Blue.

In this study, we aimed to accumulate kiwifruit-derived actinidin in the ER lumen of *N. benthamiana*, a strategy that has proven successful for the expression of various foreign proteins in plants (Islam et al., 2019; Kim et al., 2022; Zaikova et al., 2022). To achieve ER localization, we incorporated the N-terminal signal peptide of binding immunoglobulin protein (BiP) and the C-terminal ER retention signal, HDEL, into the MCS-Actinidin construct (**Figure 1A**). Additionally, the M domain of the human receptor-type tyrosine-protein phosphatase C was included to enhance translational efficiency and stability of ER-localized proteins (Kang et al., 2018). For affinity purification, the CBM3 tag, which binds irreversibly to MCC beads, was added to the construct (**Figure 1A**) (Islam et al., 2019). Since the N-terminal region containing the BiP signal sequence, M domain, and CBM3 tag is unnecessary for actinidin activity post-purification, a bdSUMO domain was included before the actinidin-coding sequence to facilitate its removal. The bdSUMO domain enables specific cleavage by His:bdSENP1 protease, which recognizes the bdSUMO sequence and cleaves after the conserved two glycine residues (Islam et al., 2019). To ensure high expression levels, the expression cassette was driven by the cauliflower mosaic virus 35S promoter and terminated with the HSP transcriptional terminator (**Figure 1A**) (Nagaya et al., 2010).

### Expression and purification of actinidin from *N. benthamiana* leaves

**Figure 2:**
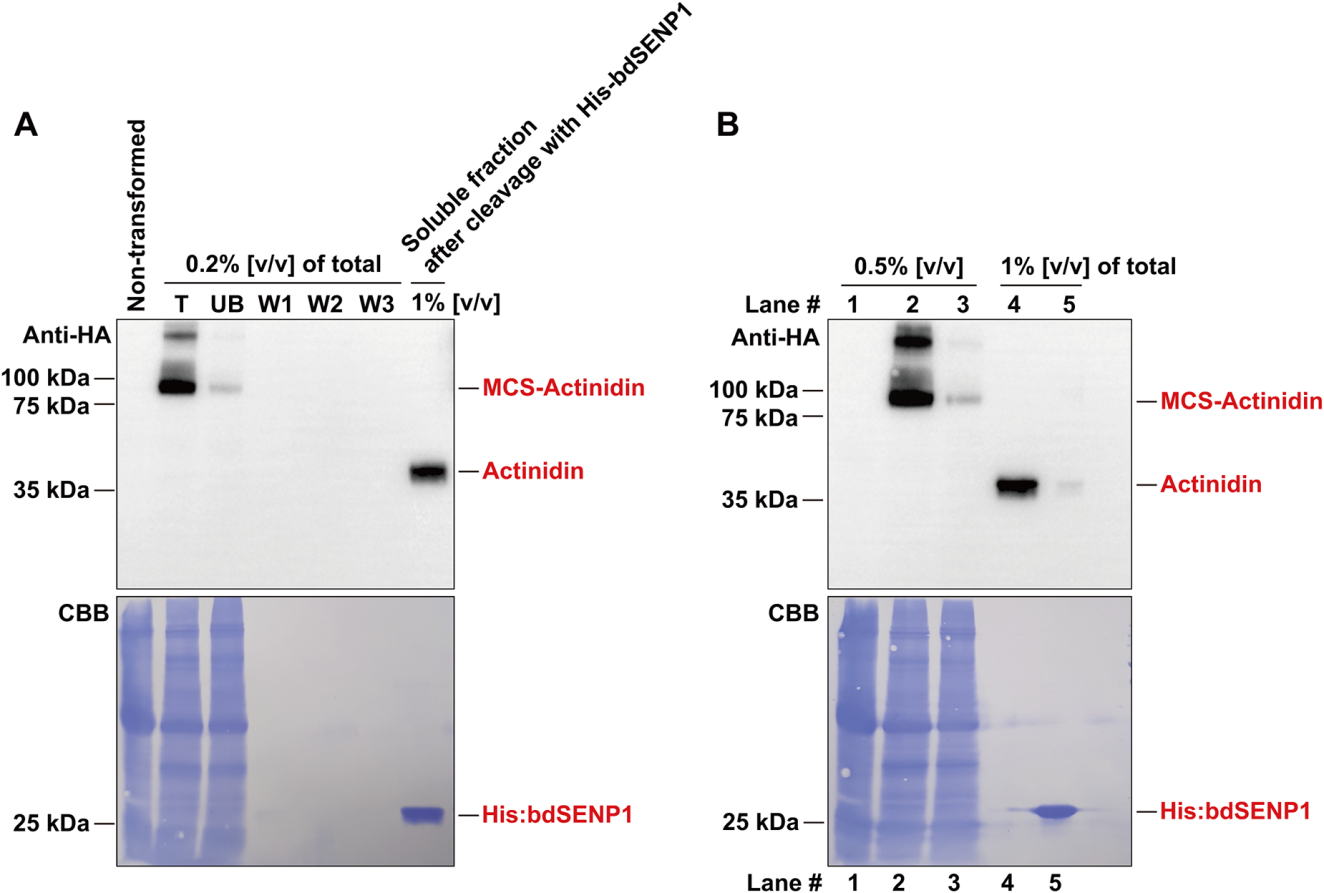
Removal of M, CBM3, and bdSUMO domains by His:bdSENP1 protease. **(A)** The purified MCS-Actinidin (**Figure 1C**) was incubated with His:bdSENP1 protease, which was purified from *E. coli*. After the reaction, the removal the domains upstream of actinidin was confirmed by Western blotting with an anti-HA antibody. Proteins were resolved on a 10% SDS-PAGE gel. CBB, Coomassie Brilliant Blue. **(B)** Removal of His:bdSENP1. The supernatant fraction from **(A)** was passed through a Ni^2+^-NTA column to remove His:bdSENP1. Proteins were resolved on a 10% SDS-PAGE gel. Lane 1, non-transformed control; Lane 2, total fraction; Lane 3, unbound fraction; Lane 4, supernatant fraction after His:bdSENP1 removal; Lane 5, fraction bound to Ni^2+^-NTA column. CBB, Coomassie Brilliant Blue.

The *MCS-Actinidin* construct was introduced into *N. benthamiana* leaves via agro-infiltration (Islam et al., 2019). At 3, 5, and 7 days post-infiltration, total protein extracts were prepared and subjected to centrifugation. The total, soluble, and pellet fractions were analyzed by Western blotting with an anti-HA antibody (**Figure 1B**). The majority of the expressed MCS-Actinidin was found in the soluble fraction, which facilitated the subsequent affinity purification process (**Figure 1B**). MCS-Actinidin was then purified from the soluble fraction using MCC beads (**Figure 1C**) (Islam et al., 2019). To remove the domains upstream of actinidin, the MCS-Actinidin conjugated to MCC beads was incubated with His:bdSENP1 protease, which recognizes the SUMO domain and cleaves immediately after Gly-Gly motif (**Figures 1A**, **2A**) (Islam et al., 2019). After the cleavage reaction, the His:bdSENP1, which was no longer necessary, was removed using Ni^2+^-NTA beads (**Figure 2B**) (Islam et al., 2019). The yield of purified actinidin was approximately 10 mg per kg of fresh *N. benthamiana* leaves.

### Actinidin expressed in *N. benthamiana* effectively degrades the substrate β-casein

**Figure 3:**
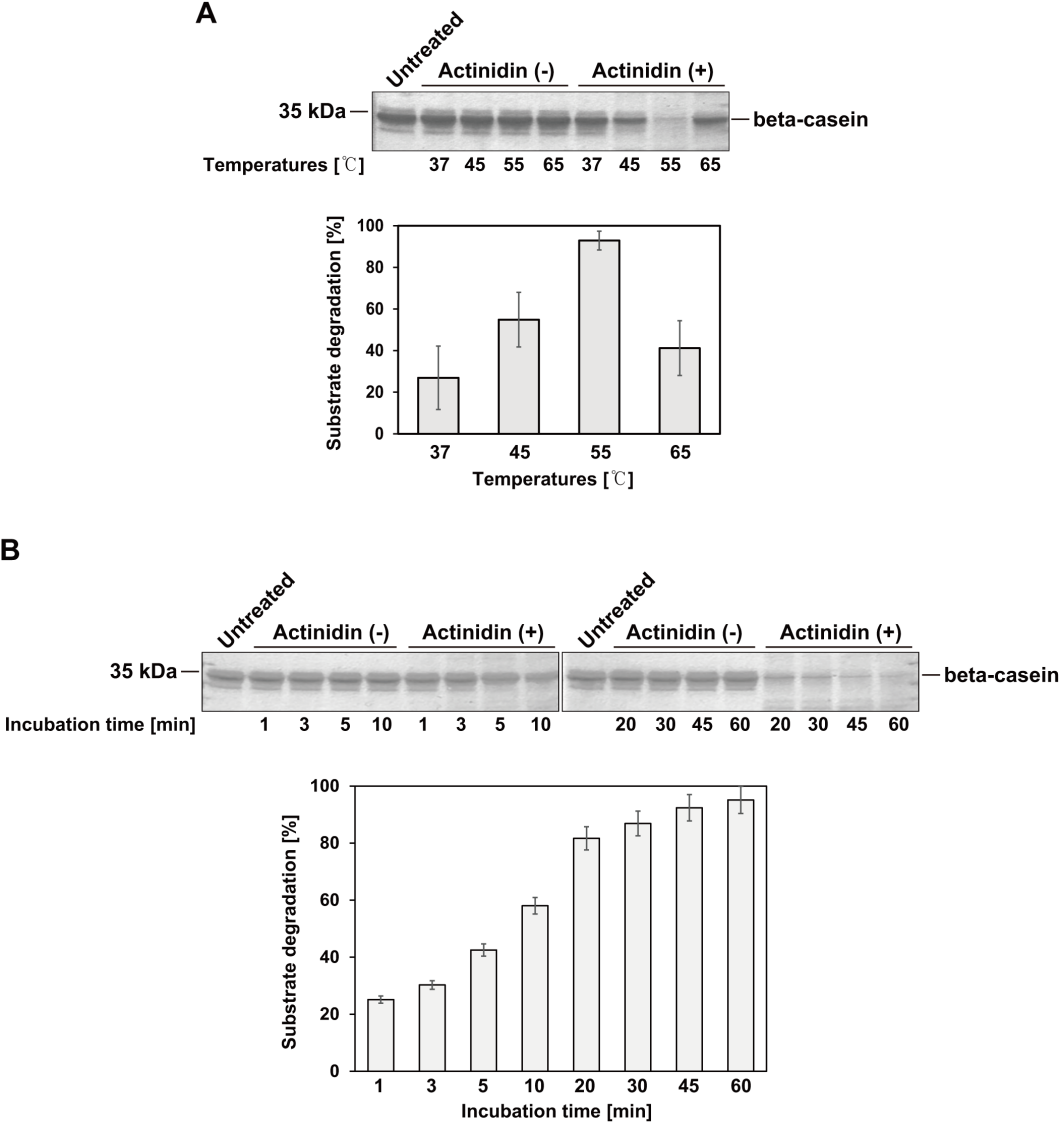
Activity of actinidin expressed in *N. benthamiana.* **(A)** The substrate β-casein (7 μg) was incubated in the absence (-) or presence (+) of purified actinidin (70 ng) at the indicated temperatures and pH 7.0 for 1 hour. After incubation, the protein samples were subjected to SDS-PAGE followed by Coomassie Brilliant Blue (CBB) staining. Proteins were resolved on a 15% SDS-PAGE gel. Band intensities were measured using ImageJ software. The relative band intensities of β-casein in the presence of actinidin (+) compared to the absence (-) at the same temperature were quantified to assess substrate degradation efficiency. **(B)** The substrate β-casein (7 μg) was incubated in the absence (-) or presence (+) of purified actinidin (70 ng) at 55°C and pH 7.0 for varying durations. Proteins were resolved on a 15% SDS-PAGE gel. Following incubation, protein samples were analyzed by SDS-PAGE and stained with CBB. Band intensities were measured using ImageJ software. The relative band intensities of β-casein in the presence of actinidin (+) compared to the absence (-) were quantified to determine substrate degradation efficiency over time.

β-casein is a well-known substrate for kiwifruit-derived actinidin (Lo Piero et al., 2011; Puglisi et al., 2012). To verify the activity of actinidin expressed in *N. benthamiana*, β-casein was incubated with or without the purified enzyme at 37, 45, 55, and 65°C for 1 hour (**Figure 3A**). The purified actinidin exhibited the highest activity at 55°C (**Figure 3A**). Next, β-casein was incubated with or without the purified actinidin at 55°C for varying durations, ranging from 1 to 60 minutes (**Figure 3B**). Compared to the untreated control, β-casein was progressively degraded as the incubation time increased (**Figure 3B**).

## Discussion

The heterologous expression of foreign proteases presents a significant challenge due to their ability to cleave peptide bonds in various intracellular proteins, potentially disrupting cellular metabolism (Luniak et al., 2017). In this study, we successfully expressed kiwifruit-derived actinidin, a cysteine protease, in *N. benthamiana* leaves, as no expression platform for actinidin has been established despite its diverse applications. The actinidin was targeted to the ER lumen, which provides an optimal environment for protein folding and post-translational modifications essential for functional protein expression. Notably, a previous study demonstrated the successful expression of the full-length adipose triglyceride lipase (ATGL) enzyme in *N. benthamiana* – a protein that could not be efficiently expressed in *E. coli*, mammalian, or insect cells (Kulminskaya et al., 2021; Zaikova et al., 2022). These findings highlight that a transient expression system utilizing *N. benthamiana* and the ER lumen as a subcellular organelle for protein accumulation provides a robust platform for the production of difficult-to-express proteins. Another potential reason for the successful expression of actinidin in *N. benthamiana* could be the plant-specific posttranslational modifications (PTMs) that occur in the ER (Gomord and Faye, 2004; Singh et al., 2021). Given that actinidin originates from kiwifruit, a plant species, it is plausible that PTMs such as glycosylation in the ER of *N. benthamiana* contribute to the solubility, stability, and functionality of the expressed actinidin. MCS-Actinidin was purified using MCC beads, which irreversibly bind to the CBM3 tag (Islam et al., 2019). The upstream domains of actinidin were then removed by His-bdSENP1 protease, which specifically cleaves immediately after the SUMO domain. This purification strategy has been successfully applied to various proteins, including enzymes, growth factors, cytokines, and vaccines (Islam et al., 2019, 2020; Razzak et al., 2020; Geem et al., 2021; Kim et al., 2022; Zaikova et al., 2022). The protein yield (approximately 10 mg/kg of fresh mass from transformed leaves) remains insufficient for industrial applications. To scale up actinidin production, several strategies could be considered, including the establishment of stable transgenic *N. benthamiana* lines and incorporating viral replicons into the vector to enhance transformation efficiency (Kim et al., 2024). However, as mentioned above, because actinidin is a protease, it is possible that its expression may adversely affect the growth of transgenic *N. benthamiana* by targeting cellular proteins. The purified actinidin exhibited temperature-dependent β-casein-degrading activity. Consistent with previous findings, activity increased with temperature, peaking at 55°C, but dropped dramatically at 65°C, likely due to protein denaturation (**Figure 3A**) (Homaei and Etemadipour, 2015). Taken together, these results suggest that *N. benthamiana*-expressed actinidin, derived from kiwifruit, holds significant potential for applications in food processing industries, such as milk clotting or meat tenderization, leveraging its proteolytic properties.

## Data Availability

The original contributions presented in the study are included in the article/supplementary material. Further inquiries can be directed to the corresponding author.
